# Editorial: Highlights in psychology of aging: non-pharmacological interventions for people at risk of or living with dementia

**DOI:** 10.3389/fpsyg.2024.1359171

**Published:** 2024-03-26

**Authors:** Mario Bernardo-Filho, Danúbia da Cunha de Sá-Caputo

**Affiliations:** ^1^Laboratório de Vibrações Mecânicas e Práticas Integrativas, Departamento de Biofísica e Biometria, Instituto de Biologia Roberto Alcantara Gomes and Policlínica Universitária Piquet Carneiro, Universidade do Estado do Rio de Janeiro, Rio de Janeiro, Brazil; ^2^Instituto Saúde.com LTDA, Rio de Janeiro, Brazil

**Keywords:** dementia, non-pharmacological interventions, aging, cognitive functions, psychological symptoms

## Introduction

Aging is a natural, complex, and multifactorial phenomenon (Silva et al., 2023) that can be associated with a healthy or pathological progression. Moreover, aging can contribute to the degradation of cognitive functions, which leads to the onset of dementia. Dementia is the seventh leading cause of death across the world. It is caused by various types of neurodegenerative disorders, with Alzheimer's disease (AD), vascular dementia, dementia with Lewy bodies, and frontotemporal dementia being the most common underlying diseases (Reynolds et al., 2022; Lin et al., 2023; Silva et al., 2023; World Health Organization, 2023).

It is reported that dementia has troublesome physical, psychological, social, and economic impacts, not only for people who are living with the condition but also for personal caregivers and families (World Health Organization, 2023). The search for effective and suitable solutions to prevent the disability and the loss of independence of older people by detecting the emerging mental, physical, and social dysfunctions is desirable and ongoing. In this context, non-pharmacological (Sá-Caputo et al., 2023) and pharmacological interventions are available for the management of individuals with dementia aiming at a potential delay of the deterioration of cognitive functions. Unfortunately, pharmacological therapy has limited effects on alleviating the behavioral and psychological symptoms of dementia (Dyer et al., 2018) and carries undesirable side effects (Buckley and Salpeter, 2015). Consequently, non-pharmacological interventions have been attracting increasing scholarly interest and are more desirable for improving or maintaining functional capacity, decreasing responsive behaviors, and reducing emotional disorders. Moreover, non-pharmacological interventions (Sá-Caputo et al., 2023) in the management of physical and/or mental impairments in older individuals are preferable as they can be considered minimally invasive, effective, and generally require low cost.

The current Research Topic *Frontiers in Psychology*—*Highlights in psychology of aging: non-pharmacological interventions for people at risk of or living with dementia* offers readers a selection of articles that cover a wide range of non-pharmacological interventions for people at risk of or living with dementia. Moreover, these articles represent the broad diversity of non-pharmacological intervention research performed across the field of “psychology of aging.” Moreover, data presented in these articles give a better understanding of some of the non-pharmacological interventions, promoting evidence-based clinical practice. The Research Topic includes eight publications addressing scientific contributions related to non-pharmacological interventions in individuals with dementia: two reviews (one scoping review and one mixed studies systematic review with narrative synthesis, thematic synthesis, and meta-integration), one randomized controlled trial (RCT) protocol, and five original articles.

Transcranial direct current stimulation (tDCS) seems to decelerate the neurodegenerative progress and shows beneficial effects on immediate recall, learning curve, immediate verbal memory, and executive functions. An RCT evaluated the home-based tDCS in a patient with mild neurocognitive disorder (mNCD) due to possible AD. Significant desirable effects were obtained on general cognitive function, immediate and delayed memory and learning ability, with an increase in scores in the active tDCS group (Satorres et al.).

In a prospective cohort study, the association of doing housework with the risk of dementia among participants aged older than 65 years was evaluated as part of the Chinese Longitudinal Healthy Longevity Survey (CLHLS) due to the relevance of physical activity (PA) to improve physical functioning and mental health and to reduce the incidence of dementia. It was observed that a high frequency of performing housework was associated with a reduced incidence of dementia among older Chinese adults (Wang et al.).

Participation in psychosocial enrichment activities, such as music and arts programming, has shown the potential to delay or reduce functional decline without adverse effects. The performing arts programming described in a community case study was inspired by a community music program called B-Sharp Music Wellness, conducted in Phoenix, Arizona, which involved small groups of musicians who provided symphony performances for people with dementia. Program outcomes suggest strategies and beneficial effects for the design of performing-arts programs as psychosocial interventions in other communities (Malinin et al.).

Photobiomodulation (PMB) is a non-invasive stimulation technique that uses light from the red to near-infrared spectrum. When PBM is applied on the scalp with the aim of influencing the brain through the skull, this procedure is called tPBM. A case study evaluated if tPBM could improve the frontal lobe cognitive functions and mental health in some older adults with mild neurocognitive disorder (mNCD). The potential effect of tPBM to improve the frontal lobe cognitive functions and mental health of older adults with mNCD was demonstrated (Cheung et al.).

Light therapy (LT) uses artificial light exposure. As it is believed that LT could mitigate the symptoms of dementia, a pilot study was performed to understand the effects of bright LT on neuropsychiatric behaviors and cognitive function in older people with dementia. It was observed that exposure to bright light led to an improvement in Neuropsychiatric Inventory scores (Liu et al.).

Social activity is highly relevant to the prevention of cognitive decline. However, providing real-time social interaction as a non-pharmacological intervention is possible only in limited circumstances has reduced availability. Miura et al. developed the Photo-Integrated Conversation Moderated by Application (PICMOA), a home-based group conversation intervention using smartphones. In an RCT protocol, the PICMOA intervention was introduced to evaluate effects of PICMOA on the cognitive functioning and psychological wellbeing of Japanese community-dwelling older adults at the risk of cognitive function decline. The relevance of the PICMOA intervention would be due to the interest growing in internet-based activities for preventing social isolation, but the effect of remote conversation interventions on cognitive functioning remains unclear. This study would provide a new avenue for the social participation for older adults (Miura et al.).

Music-based interventions, including sensory stimulation, would be the first-choice approach for managing the behavioral and psychological symptoms of dementia. Low-frequency (LF) sinusoidal mechanical vibration interventions related to music interventions may offer relief for these symptoms. It is known that interest in the effectiveness of auditory music interventions and music therapy for managing individuals with dementia has increased, but LF vibration has not been included. A scoping review was conducted to investigate participants' responses to both sound and mechanical vibration. It was concluded that higher quality research is needed to investigate the impact and effect of LF vibration on dementia symptoms (Campbell et al.).

Nevertheless, considering that the efficacy of music-based interventions for people with dementia has focused on specific outcomes and methods, and singing has been noted as a particularly beneficial activity, Thompson et al. described in a mixed studies systematic review with narrative synthesis, thematic synthesis, and meta-integration “how singing can help people with dementia and their family care-partners.” It is suggested that singing could positively impact the lives of people with dementia and their caregiving partners. Due to the heterogeneity of the study design and outcome measures, it was difficult to draw conclusions based on quantitative data alone. Nonetheless, qualitative data provides insights from the participant perspectives, and when integrated with quantitative data, demonstrates the benefits of singing.

## Conclusion

Dementia is one of the major causes of disability and dependency among older people globally. In 2020, ~50 million people were living with dementia globally, and this number is expected to reach 152 million by 2050 (Livingston et al., 2020). Moreover, it is known that the number of older individuals is increasing worldwide. In this context, knowledge about non-pharmacological interventions as a strategy to improve the quality of life of individuals with dementia is desirable and necessary. Moreover, non-pharmacological interventions can be considered minimally invasive, effective for the management of dementia individuals, and generally require low cost. In the current Research Topic, studies about (i) home-based tDCS, (ii) housework as a type of PA, (iii) tPBM, (iv) bright LT, (v) PICMOA, (vi) arts, (vii) LF mechanical vibration, and (viii) singing are presented.

## Author contributions

MB-F: Validation, Supervision, Methodology, Writing—review & editing, Writing—original draft, Funding acquisition, Conceptualization. DS-C: Writing—review & editing, Validation, Supervision.
